# Inguinal bladder hernia treated using transabdominal preperitoneal approach: A case report

**DOI:** 10.1016/j.ijscr.2019.11.045

**Published:** 2019-11-27

**Authors:** Yosuke Namba, Toshikatsu Fukuda, Syo Ishikawa, Azusa Kai, Akihiro Kohata, Syo Okimoto, Shoichiro Mukai, Seiji Fujisaki, Yuzo Hirata, Saburo Fukuda, Mamoru Takahashi

**Affiliations:** a Department of Surgery, Chugoku Rosai Hospital, Japan; b Department of Gastroenterological and Transplant Surgery Applied Life Sciences, Institute of Biomedical and Health Sciences, Hiroshima University, Japan

**Keywords:** IBH, inguinal bladder hernia, TAPP, transabdominal preperitoneal approach, CT, computed tomography, Inguinal hernia, Bladder hernia, Urinary bladder, Transabdominal preperitoneal approach

## Abstract

**Introduction:**

Inguinal bladder hernia (IBH) is a rare condition that is difficult to diagnose preoperatively based only on physical examination; 16% of IBHs are diagnosed postoperatively due to complications.

**Presentation of case:**

We report the case of a 56-year-old man who presented with left inguinal swelling and increased frequency of urination since eight years. Physical examination demonstrated a 6 × 4 cm non-reducible left inguinal bulge with mild tenderness on palpation. Computed tomography revealed a left inguinal hernia containing a portion of the urinary bladder. He was diagnosed with IBH and transabdominal preperitoneal (TAPP) repair was performed. We confirmed a left internal inguinal hernia and incised the peritoneum from the outside of the left inguinal ring. The preperitoneal space was dissected toward the Retzius space, and the prolapsed bladder was examined. The adhesion with the surroundings was carefully dissected and the bladder was reduced into the abdomen. Indigo carmine was injected through a urinary catheter, which confirmed that no bladder damage had occurred. After the mesh was positioned to cover the myopectineal orifice, it was fixed to the Cooper’s ligaments, interior side, lateral side, and ventral side. The postoperative course was uneventful, and the patient is now free of symptoms and recurrence two months after surgery.

**Conclusion:**

TAPP repair is a useful treatment for IBH. Preoperative diagnosis of IBH is important to lessen postoperative complications.

## Highlights

• Inguinal bladder hernia (IBH) is a rare condition that is difficult to diagnose preoperatively.
• 16% of IBHs are diagnosed postoperatively due to bladder injury and leakage.
• Preoperative diagnosis of IBHs is important to lessen postoperative complications.
• Transabdominal preperitoneal approach can significantly reduce the risk of bladder damage.

## 1 Introduction

Inguinal bladder hernia (IBH) is a rare condition that was first described by Levine in 1951 [1]. IBHs are found in 1–4% of inguinal hernias in the general population; in obese men above the age of 50 years, this incidence approaches 10% [2,3]. IBHs occur through an acquired peritoneal defect, classifying it as a direct inguinal hernia and therefore increasing in incidence with age [2]. Other risk factors include male sex, obesity and chronic urinary obstruction [4]. Diagnosis of IBHs with only physical examination is difficult because the majority of patients are asymptomatic; therefore, diagnosis is confirmed with ultrasonography or radiography [5]. Only 7% of IBHs are diagnosed prior to surgery, 16% are diagnosed postoperatively due to complications including bladder injury and leakage, and the rest are diagnosed intraoperatively [6]. Preoperative diagnosis of IBHs is important to lessen postoperative complications. Along with preoperative imaging, the transabdominal preperitoneal approach (TAPP) is also useful to reduce complications. In TAPP, the operator can recognize the type of hernia and the operation can be performed safely. Moreover, we can confirm the presence or absence of bladder damage by using indigocarmine during surgery. In this report, we describe the case of a left IBH treated using TAPP repair. This work has been reported in line with the SCARE criteria [7].

## 2 Presentation of case

**Fig. 1:**
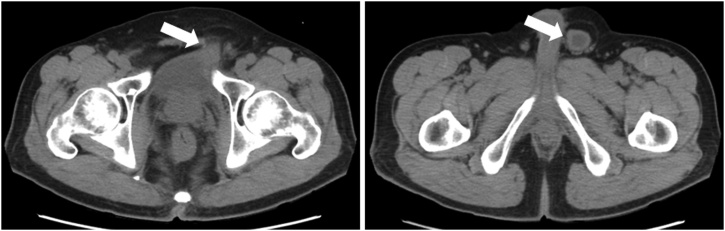
Computed tomography (CT). CT revealed a left inguinal hernia containing a portion of the urinary bladder (arrow).

A 56-year-old man presented to our hospital with left inguinal swelling and increased frequency of urination lasting eight years. He had no significant medical or surgical history. Physical examination demonstrated a non-reducible 6 × 4 cm left inguinal bulge with mild tenderness on palpation. Computed tomography (CT) revealed a left inguinal hernia containing a portion of the urinary bladder (Fig. 1). He was diagnosed with IBH and TAPP repair was performed.

**Fig. 2:**
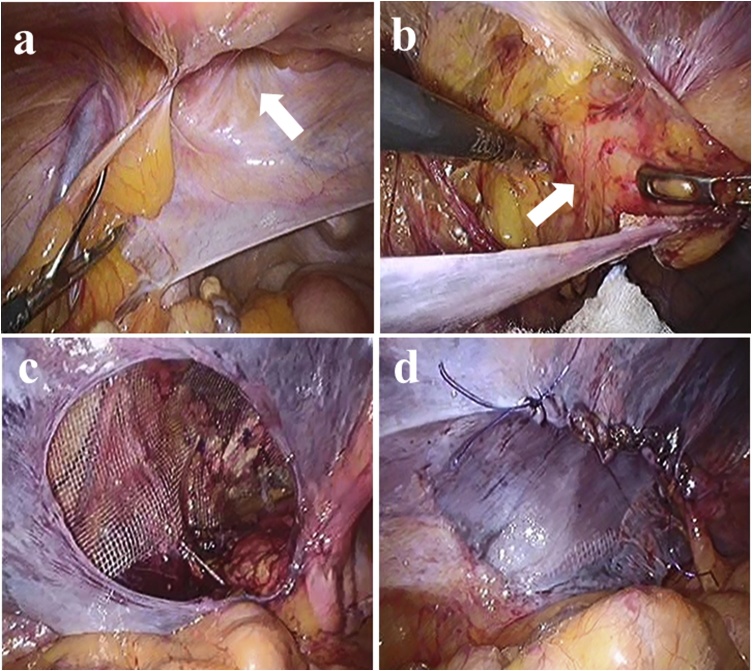
transabdominal preperitoneal approach. a. A left internal inguinal hernia was confirmed (arrow). b. The bladder had a strong adhesion with the surroundings and it was in a state of incarceration (arrow). c. The mesh was positioned to cover the Hesselbach triangle and left inguinal ring, it was fixed to the Cooper’s ligaments, interior side, lateral side, and ventral side. d. The peritoneal defect was closed with a running suture.

During the laparoscopic surgery, the patient was placed in the supine position under general anesthesia. A urinary catheter was inserted before the operation. A 5 mm initial trocar was inserted into the umbilicus with the optical method, and carbon dioxide was insufflated at 10 mmHg. At first, we confirmed a left internal inguinal hernia (Fig. 2a). Two 5 mm trocars were placed in each flank, and we incised the peritoneum from the outside of the left inguinal ring. The preperitoneal space was dissected toward the Retzius space, and the prolapsed bladder was checked. The bladder had developed strong adhesions with the surroundings and it was in a state of incarceration (Fig. 2b). The adhesions were carefully dissected and the bladder was reduced into the abdomen. Indigo carmine was injected through the urinary catheter, which confirmed the absence of bladder damage. The ventral and lateral sides were dissected to secure a space for the mesh. A 15 × 10 cm TiLENE mesh (PFM Medical, Koln, Germany) was inserted through the incision. After the mesh was positioned to cover the myopectineal orifice, it was fixed to the Cooper’s ligaments, interior side, lateral side, and ventral side using 5 mm AbsorbaTac (Medtronic company, COVIDIEN, Tokyo, Japan) (Fig. 2c). The peritoneum was closed with a running suture with 4-0 PDSII (polydioxanone Ethicon, Ltd, USA) (Fig. 2d). The total procedure time was 86 min, and blood loss was 1 g.

The patient’s urinary catheter was removed immediately after the surgery and the patient was able to urinate without pain or difficulty. The patient was discharged to his home on postoperative day 3 without recurrence or other complications. His urinary frequency improved on postoperative day 10.

## 3 Discussion

IBHs occur in 1–4% of all inguinal hernias, and its prevalence approaches 10% in obese men above the age of 50 years [2,6]. Studies from 2004 have shown that 11.2% of IBHs were associated with urologic malignancies and 23.5% were associated with a various complications [8,9].

The pathophysiology of IBHs may be related to pulling of the bladder together with a sheath of peritoneum that forms its sac, through a weak point in the abdominal fascia [8,10]. Some factors including bladder outlet obstruction, obesity, decreased bladder tone, and weakness of the pelvic musculature are associated with the development of IBHs [[11], [12], [13]]. Some reports state that IBHs are more common on the right side; left sided hernias, as seen in our case are rare [11]. IBHs are usually asymptomatic, but may present with nonspecific symptoms including urinary frequency, urgency, nocturia, and hematuria [14]. For example, our patient had an increased urinary frequency of about once an hour.

Preoperative diagnosis is very important for IBHs. Khan et al. reported that only 7% of IBHs are diagnosed prior to surgery, 16% are diagnosed postoperatively due to complications including bladder injury and leakage, and the rest are diagnosed intraoperatively. Preoperative diagnosis may reduce the potential complications of IBHs. Medical history and physical examination are important for the preliminary diagnosis; ultrasonography, cystography, and CT are utilized to confirm the diagnosis. Diagnosis should be made extra carefully in obese men above the age of 50 years as they have a 10% likely incidence of IBHs. Additionally, TAPP repair may be a factor in reducing complications. In TAPP, prolapsed organs can be confirmed through intraperitoneal or preperitoneal observation, and the presence or absence of bladder damage can be confirmed by injecting indigo carmine through the urinary catheter. Because the operation is performed in the Retzius space, dissection can be performed while checking the bladder. For these reasons, TAPP repair can significantly reduce the risk of bladder damage. We should perform TAPP repair after finalizing the diagnosis, because the hernia cannot be confirmed by examining the intraperitoneal space in the extraperitoneal type of IBH [15].

In this case, we diagnosed IBH before surgery by studying the medical history, physical examination, and CT. We performed TAPP repair and proceeded without any complications during and after surgery.

## 4 Conclusion

We report the case of a patient who underwent successful TAPP repair for IBH. TAPP procedure is a useful treatment for IBH. Preoperative diagnosis of IBH is important to lessen postoperative complications.

## Funding

No source of funding to be declared.

## Ethical approval

Ethical approval was not required and patient identifying knowledge was not presented in the report.

## Consent

Written informed consent was obtained from the patient for publication of this case report and accompanying images.

## Author contribution

Yosuke Namba: Managed the patient and drafted the manuscript.

Toshikatsu Fukuda: Managed the patient, supervised the writing of the manuscript, and approved the final manuscript.

Sho Ishikawa, Akinori Kohata, Azusa Kai, Yuzo Hirata, Seizi Fuzisaki, Saburo Fukuda, Mamoru Takahashi: Conception and design.

## Registration of research studies

Our study does not require registration.

## Guarantor

Toshikatsu Fukuda.

## Provenance and peer review

Not commissioned, externally peer-reviewed.

## Declaration of Competing Interest

The authors declare that they have no conflicts of interest.
